# Carcinoma of the anal canal: Intensity modulated radiation therapy (IMRT) versus three-dimensional conformal radiation therapy (3DCRT)

**DOI:** 10.1002/jmrs.28

**Published:** 2013-11-25

**Authors:** Charlotte Sale, Phillip Moloney, Maitham Mathlum

**Affiliations:** Andrew Love Cancer Centre, Geelong HospitalGeelong, Victoria, Australia

**Keywords:** Anal carcinoma, IMRT, three-dimensional conformal radiation therapy

## Abstract

**Introduction:**

Patients with anal canal carcinoma treated with standard conformal radiotherapy frequently experience severe acute and late toxicity reactions to the treatment area. Roohipour et al. (*Dis Colon Rectum* 2008; **51**: 147–53) stated a patient's tolerance of chemoradiation to be an important prediction of treatment success. A new intensity modulated radiation therapy (IMRT) technique for anal carcinoma cases has been developed at the Andrew Love Cancer Centre aimed at reducing radiation to surrounding healthy tissue.

**Methods:**

A same-subject repeated measures design was used for this study, where five anal carcinoma cases at the Andrew Love Cancer Centre were selected. Conformal and IMRT plans were generated and dosimetric evaluations were performed. Each plan was prescribed a total of 54 Gray (Gy) over a course of 30 fractions to the primary site.

**Results:**

The IMRT plans resulted in improved dosimetry to the planning target volume (PTV) and reduction in radiation to the critical structures (bladder, external genitalia and femoral heads). Statistically there was no difference between the IMRT and conformal plans in the dose to the small and large bowel; however, the bowel IMRT dose–volume histogram (DVH) doses were consistently lower.

**Conclusion:**

The IMRT plans were superior to the conformal plans with improved dose conformity and reduced radiation to the surrounding healthy tissue. Anecdotally it was found that patients tolerated the IMRT treatment better than the three-dimensional (3D) conformal radiation therapy. This study describes and compares the planning techniques.

## Introduction

Anal canal carcinomas are relatively rare. There are approximately 300 new cases each year in Australia.^1^ The incidence of anal carcinoma is increasing in Australia, similar to international figures.^1^,^2^ A combination of radiation therapy and chemotherapy has been the standard treatment since 1974,^3^ with greater than 70% colostomy-free survival rates reported by Milano et al.^4^ and 5 years ranging between 50% and 88% depending on the stage of disease. However, the radiation dose and technique for radiation delivery remain a topic of debate.^5^,^6^

**Figure 1:**
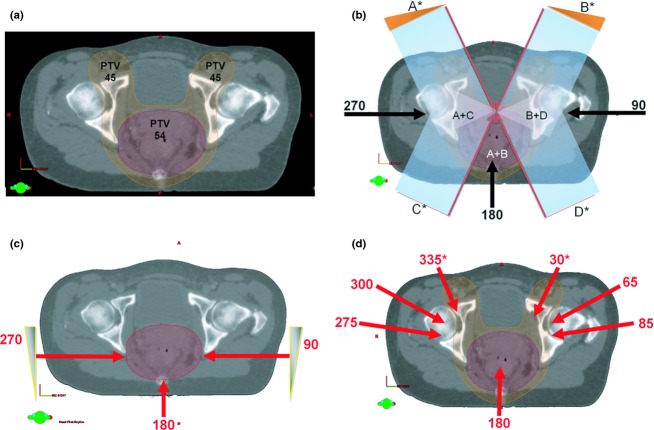
(a) Transverse view of the unique shape of the planning target volume (PTV) surrounding the critical structures. The PTV45 is represented by the orange region and the PTV54 by the red. (b) The seven-field conformal beam arrangement (Phase 1). The beams for the conformal technique are 30°, 90°, 155°, 180°, 210°, 270° and 335°. Those angles marked with an asterisk (*) have been customized for each patient to follow the medial edge of the PTV45 (illustrated in orange). (c) The three-field conformal beam arrangement (Phase 2). The field angles comprised of a posterior field with 6 MV and two lateral fields with 18 MV. (d) The Andrew Love Cancer Centre IMRT beam arrangement. The beams for the conformal technique are 30°, 65°, 85°, 180°, 275°, 300° and 335°. Those angles marked with an asterisk (*) have been customized for each patient to follow the medial edge of the PTV45 (illustrated in orange).

The size and shape of the anal canal has historically presented challenges when planning conformal radiation therapy. The planning target volume (PTV) forms an elongated horseshoe shape that surrounds, but does not encompass, the healthy tissue (bladder, genitals and bowel) within (refer to Fig. 1a). The radiation dose delivered to the tumour volume is limited by the healthy tissue surrounding the PTV and the complexity of the volume to cover the pelvic primary and all elective nodal groups. Maintaining a homogenous dose throughout the PTV is challenging to plan. Intensity modulated radiation therapy (IMRT) was proposed at the Andrew Love Cancer Centre in 2010 to improve the dosimetry and ease of planning for these cases.

Intensity modulated radiation therapy with image-guided radiation therapy (IGRT) and clearly defined volumes of interest has the potential to improve tumour control and reduce acute and late toxicities with subsequent improvement in quality of life (QOL). Traditional (conformal) radiation therapy techniques with chemotherapy have resulted in significant acute and late morbidity.^7^ Acute side effects experienced include severe skin reaction, proctitis, dysuria, bone marrow suppression, diarrhoea, perineal and anal canal pain, and vaginal mucositis.^6^,^8^,^9^ Long-term side effects include perineal skin atrophy, fibrosis, vaginal stenosis and dryness, sexual dysfunction, anal sphincter dysfunction, chronic proctitis and femoral neck fracture.^9^,^10^ Although there have been publications on the use of IMRT for anal carcinoma treatment over the past decade, this study describes the unique IMRT anal canal technique developed at the Andrew Love Cancer Centre aimed at reducing the dose to the surrounding healthy tissue to reduce toxicity.

Previous studies on anal carcinoma treatment have investigated various techniques for both conformal and IMRT planning.^4^,^11^,^12^ Milano et al.^4^ compared an anteroposterior/postero-anterior (AP-PA) conformal technique with a nine-field IMRT technique. It was considered that it should be relatively easy to achieve improved dosimetry when comparing a simple two-field technique with a nine-field IMRT plan. Menkarios et al.^11^ went further to compare a conformal plan (AP-PA with a three-field boost) with three different IMRT plans. Most recently, Brooks et al.^12^ compared a two-phase, four-field conformal technique that was the most sophisticated conformal plan to be reported in the literature to date. However, the conformal technique in these studies^4^,^11^,^12^ remained a relatively simple plan in comparison with the traditional conformal plan used at the Andrew Love Cancer Centre (refer to Fig. 1b and c). The IMRT plans described by Menkarios et al.^11^ and Brooks et al.^12^ consisted of a seven-field IMRT plan, but differed in field arrangement and prescription when compared with the proposed IMRT plan at the Andrew Love Cancer Centre. Therefore, this current investigation was unique in that it was based on comparing the complex conformal anal carcinoma plan historically utilized at the Andrew Love Cancer Centre with a new IMRT technique specifically with a seven-field beam arrangement.

The aim of this study was to investigate the effect of the new IMRT technique on the dose received by the PTV and critical structures, compared with the historical conformal technique for patients with anal canal cancer. Although statistically significant improvements with the newer planning technique were aimed for, clinically significant improvements were also viewed as important. Although the dosimetric benefits of this new IMRT technique were clear, this study quantifies the evidence to support the use of the new IMRT technique over the historical conformal technique. This study will describe the two planning techniques and present a comparison of the two methods with dosimetry analysis. The research questions were as follows:

1. Does the IMRT technique used at the Andrew Love Cancer Centre improve the dosimetric coverage of the tumour volume when compared with the historical conformal technique?
2. Does the IMRT technique reduce the dose received by surrounding critical structures compared with the historical conformal technique?

## Methods

### Research design

A same-subject repeated measures design was used for this study. Five anal carcinoma cases at the Andrew Love Cancer Centre were selected for this study. Prior to April 2010, there were three cases planned and treated with a conformal technique. In April 2010, two patients with carcinomas of the anal canal presented and received the new IMRT treatment. The five patients' computed tomography (CT) data sets were planned with conformal and IMRT techniques. Due to the rarity of this disease, five cases, which equated to 10 plans, were viewed as being reasonable to indicate differences between the planning techniques. Observed power and the sample size required (*α* = 0.05, *β* = 0.8) to achieve statistical significance were calculated for each comparison. The dose distribution throughout the PTV and the critical structures were compared, using dose–volume histogram (DVH) data and two conformity indices. General observations from the radiation oncology team (radiation oncologists, radiation therapists, and oncology nurses) on the patients' tolerance of the treatment, as well as available toxicity data forms part of the Discussion.^13^

### CT simulation

To minimize the effect of potential confounding variables between subjects, a same-subject approach was used in this study, where the dose distribution was compared on the same patient's CT data set for each of the planning techniques (IMRT and conformal forward planning). All planning was completed on CT data sets constructed using 2.0-mm slices. All patients were positioned supine and scanned from above lumbar spine five (L5) to below lesser trochanter for consistency. All anal canal patients were scanned with a full bladder and no contrast. The immobilization used for these patients was the same across the cohort and included a contoured head shape to support the patients' neck, knee support and ankle fix device. All immobilization devices were indexed to the CT and treatment couch; this was to maximize the stability and reproducibility of the set-up.

### Target volume and critical structure delineation

The gross tumour volume (GTV), clinical target volume (CTV) and PTV were delineated by the radiation oncologist on the transverse slices of the CT data set. These target volumes were delineated in accordance with the International Commission of Radiological Units 50 guidelines.^14^ The GTV was defined as the gross primary anal tumour volume from the digital examination, CT scan, medical resonance images (MRI) and positron emission tomography (PET). The GTVp was defined as the primary site disease (anal canal). GTVn was defined as the involved lymph nodes. The CTVs were created from the GTV. The CTVa included the GTVp, the entire anal canal, plus a 1.0- to 2.0-cm expansion, except into bone or air. The CTVn included the GTVn with a 1.0-cm expansion, except into bone, air or muscle. There were also two CTVs created:

1. The CTV receiving 45 Gy (CTV45) included the CTVa and CTVn and all elective nodal groups (mesorectal, presacral lymph node, internal and external iliac lymph nodes, and inguinal lymph nodes).
2. The CTV receiving 54 Gy (CTV54) included the CTVa and CTVn (when the nodes were between 1.0 and 2.0 cm or greater).

The PTV receiving 45 Gy (PTV45) was determined by an expansion on the CTV45 with a 1.0-cm margin for internal organ motion and set-up variability. The PTV receiving 54 Gy (PTV54) was determined by expansion on the CTV54 with a 1.0-cm margin for internal organ motion and set-up variability.

Critical structures, including bladder, external genitalia, femoral heads, and small and large bowel, were delineated on the CT data set, as solid structures by a group of three experienced radiation therapist in planning, then reviewed and approved by the radiation oncologist. The bladder was contoured using the external outline of the bladder wall.^15^ The external genitalia was delineated to include the perineum; for females this included the clitoris, labia majora, labia minora, and skin anterior to the pubic symphysis; and for males this included the penis, scrotum and skin anterior to the pubic symphysis.^15^ The femoral heads were delineated to 1.0 cm inferior to the PTV. The small and large bowel was delineated from 1.5 cm superior to the PTV down to the rectosigmoid junction.^15^

### Radiation therapy treatment prescription

For the purpose of this study, all cases were prescribed 54 Gy in 30 fractions with two phases (Phase 1 planned to 45 Gy; and Phase 2 to 54 Gy). The target volumes and critical structure dose constraints for all planning techniques are detailed in Table 1.

**Table 1:** Dose constraints for the target and critical structures.

Structure	Dose constraint	
PTV54	Conformal^16^	IMRT^15^,^17^,^25^
	D95% ≥ 54 Gy	D98% ≥ 51.3 Gy
	D5% ≤ 57.8 Gy	D2% < 62.1 Gy
Bladder^15^	V35 Gy ≤ 50%	
	V40 Gy ≤ 35%	
	V50 Gy ≤ 5%	
External genitalia^15^	V20 Gy ≤ 50%	
	V30 Gy ≤ 35%	
	V40 Gy ≤ 5%	
Femoral heads^15^	V40 Gy ≤ 35%	
	V44 Gy ≤ 5%	
Small bowel^15^	V30 Gy ≤ 350 cc	
	V35 Gy ≤ 150 cc	
	V45 Gy ≤ 20 cc	
	V50 Gy = 0 cc	
Large bowel^26^	V55 Gy max	
	V40 Gy < 50%	

PTV54, planning target volume receiving 54 Gray; D*x*%, *x*% of the dose; VnGy, volume receiving *n* Gray (Gy); cc, cubic centimetres; max, maximum; IMRT, intensity modulated radiation therapy.

### Radiotherapy planning

This study compared the historical Andrew Love Cancer Centre conformal technique and the IMRT technique. All plans were created on the Varian Eclipse planning system, version 8.6 (Varian Medical Systems, Palo Alto, CA). Both conformal and IMRT plans can vary in quality based on the planner's skills and experience. Therefore, all planning was completed by a group of three planners, who were experienced in conformal and IMRT planning at the Andrew Love Cancer Centre. Interplanner checks took place to ensure that all plans were created using the same method and to the same degree of precision. IMRT protocol templates were also used to ensure consistency in the IMRT planning.

#### Conformal planning technique

The conformal plans used seven coplanar fields for Phase 1 with a combination of 6 and 18 megavolt (MV) photon energies. Field angles comprised of a posterior field with 6 MV photons and two lateral and four oblique fields with 18 MV. The gantry angles of the four oblique fields were customized for each patient to follow the inner face of the PTV45 horseshoe volume and to utilize common borders (refer to Fig. 1b). Common borders refer to the opposing oblique fields sharing a common border such that they each treat different regions of the PTV, which enables greater flexibility in dose optimization. The posterior field was planned with 6 MV photons due to the close proximity of the GTV to the posterior surface of the patient. Dynamic wedges were used on the anterior oblique fields. Phase 2 was forward planned using a conventional three-field coplanar technique optimized to the PTV54. Field angles comprised of a posterior field with 6 MV and two lateral fields with 18 MV (refer to Fig. 1c). Dynamic wedges were used on the lateral fields. Static multileaf collimators (MLC) were used to conform the beam to the shape of the PTV. Dose optimization subfields were created as required.

#### IMRT technique

Intensity modulated radiation therapy plans were created for the five cases using seven coplanar field angles, where opposing fields were avoided. The beams were positioned at 180°, 275°, 300°, 335°, 30°, 65° and 85° (refer to Fig. 1d). The beam angles were determined by the group of experienced planners through the use of published literature, as well as a method of ‘trial and error’ trying different beam arrangements and discussing their advantages and disadvantages. The 335° and 30° fields were customized (*) for each patient to follow the inner face of the PTV horseshoe to control dose splaying to the anterior nodal region. Six-megavolt (6 MV) photons were used for all IMRT fields. Dynamic MLCs were used to define the shape of the beams. Dose and volume constraints were defined using a protocol template (Table 2). The primary aim of the plan optimization process was to maintain the PTV dose constraints, followed by achieving the critical structure dose constraints. Optimization was completed when the PTV was encompassed by the prescribed dose; and no further sparing of the critical structures could be made without compromising PTV coverage.

**Table 2:** Optimization objectives.

Structure	Limit	Volume (%)	Gray (Gy)	Priority
Left femur	Upper	0	45.00	50
Right femur	Upper	0	45.00	50
External genitalia	Upper	0	30.00	70
IMRT bladder	Upper	50	25.00	50
IMRT bladder	Upper	5	42.00	50
IMRT bladder	Upper	25	30.00	50
Large bowel	Upper	40	33.00	50
Large bowel	Upper	60	27.00	50
Large bowel	Upper	15	35.00	50
Small bowel	Upper	20	33.00	50
Small bowel	Upper	40	27.00	50
Small bowel	Upper	50	22.00	50
IMRT PTV45	Upper	0	48.00	70
IMRT PTV45	Lower	100	47.00	70
IMRT PTV52	Upper	0	56.00	95
IMRT PTV52	Lower	100	55.00	100
Bladder	Upper	0	45.00	50
Bladder	Upper	40	30.00	50

IMRT, intensity modulated radiation therapy; PTV45, planning target volume receiving 45 Gy; PTV52, planning target volume receiving 52 Gy.

For the IMRT plans, additional volumes were created to assist the inverse planning process in producing quality plans. ‘Plus’ volumes were implemented to improve PTV coverage, whereas ‘limit’ volumes allowed the algorithm to minimize the dose to the uninvolved regions of critical structures that overlap with the PTV volume. A ‘PTV45 Plus’ structure was created by adding an expansion margin of 1.0 mm to the PTV45. ‘Limit’ structures were created excluding the regions of the bladder, external genitalia, and small and large bowel volumes that extended inside the ‘PTV45 Plus’ and the PTV54 structures with a 2.0-mm margin.

### Analysis of the planning techniques

Dose–volume histograms were generated to compare the two planning techniques (conformal and IMRT). Plan comparison DVHs were utilized to ensure that the PTV coverage was comparable for the two techniques, in accordance with the ICRU62 and ICRU83 reports.^16^,^17^ The rationale was if the PTV coverage was similar for each technique then it was reasonable to use the critical structure dosimetry for plan comparisons. In addition, dose constraint comparisons were undertaken, using DVH data of the PTV and critical structures, as indicated in Table 1. Comparisons were also made between the minimum, maximum, mean and median dosage to the PTV and critical structures. These measurements were chosen as they provided an overview of the radiation dose coverage throughout the treatment area and to the surrounding healthy tissue. The DVH data were statistically analysed, using the IBM Statistical Package for the Social Sciences (SPSS version 20; IBM Corporation, Armonk, NY). Simple descriptive statistics were used and statistical differences between the planning techniques were evaluated using a test for normality to determine if the data were normally distributed.

Two measurements were used to evaluate plan homogeneity,^18^ the Healthy Tissue Conformity Index^19^ and the conformation number^20^ and these were calculated for each plan. The Healthy Tissue Conformity Index accounted for the healthy tissue that was irradiated^19^ and is a ratio of the volume of the target within the reference isodose curve and the total volume of tissue within the reference isodose. The conformation number accounted for volume of the target irradiated, as well as the irradiated healthy tissue.

## Results

There were five patients presented and were treated for anal carcinoma at the Andrew Love Cancer Centre by the end of April 2010. These five cases were all female and ranged in age from 41 to 87 years, with a mean age of 57 years and a median age of 52 years. No cases had surgical resection prior to treatment. All cases received concurrent chemotherapy. All five patients were diagnosed with squamous cell carcinoma of the anal canal with a T staging of T2–3. Nodal involvement for this cohort was variable and ranged from N0 to N3. There were no cases with metastases. The staging for this cohort of patients ranged from II to IIIB (two cases with II, two with IIIA and one with IIIB).

**Figure 2:**
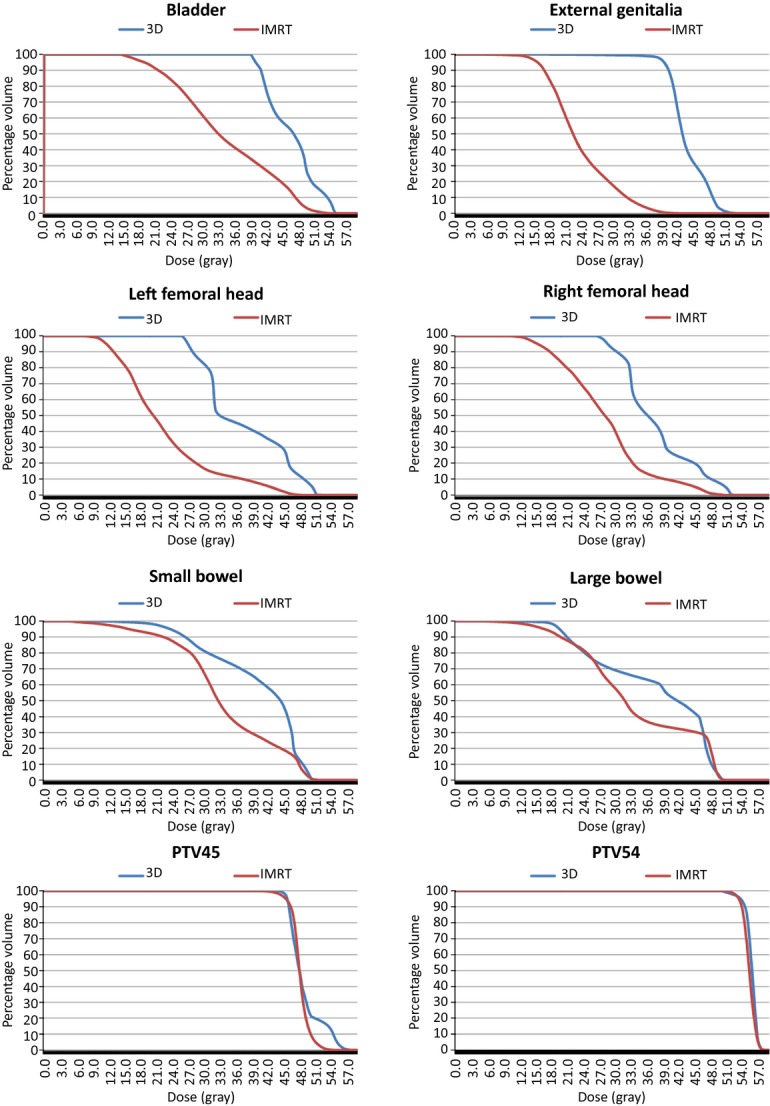
Typical cumulative dose–volume histogram data comparing the conformal and IMRT planning techniques for an individual case. 3D, conformal plan; IMRT, intensity modulated radiation therapy; PTV, planning target volume.

**Figure 3:**
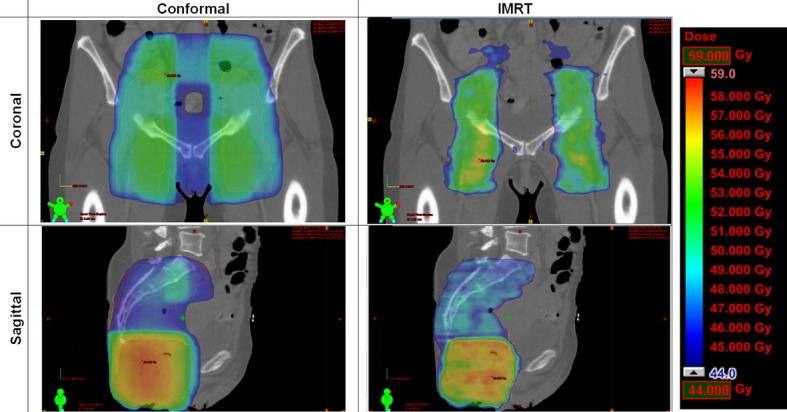
Typical dose distributions on axial sections for the conformal and intensity modulated radiation therapy (IMRT) planning techniques.

Both planning techniques (conformal and IMRT) produced adequate dosimetry to the PTV and surrounding critical structures in accordance with the criteria set in Table 1. The dose–volume data and statistical results are presented in Table 3. Typical cumulative DVH data comparing the two planning techniques for an individual case are shown in Figure 2. Typical dose distributions on axial sections for the two planning techniques (conformal and IMRT) are presented in Figure 3.

**Table 3:** Dosimetric data with significant differences between conformal and IMRT technique for anal canal cases.

Variable	Conformal technique	IMRT	Test of normality Shapiro–Wilk		*t*-Test	Observed power *β*
		
	Mean (SD)	Mean (SD)	Conformal technique	IMRT		
Normally distributed data						
PTV54						
CN	0.37 (0.03)	0.75 (0.03)	0.37	0.10	0.00^1^	1.00
HTCI	0.37 (0.02)	0.76 (0.02)	0.11	0.57	0.00^1^	1.00
Maximum	58.77 (0.90)	60.37 (0.96)	0.22	0.72	0.03^1^	0.67
Bladder						
Minimum	32.06 (7.39)	14.29 (3.68)	0.27	0.61	0.00^1^	0.99
Mean	46.01 (1.35)	33.53 (5.66)	0.93	0.16	0.00^1^	0.99
40 Gy	87.18 (11.13)	33.76 (16.20)	0.15	0.20	0.00^1^	1.00
Genitalia						
Maximum	55.38 (1.48)	45.00 (5.24)	0.58	0.33	0.00^1^	0.95
Mean	43.37 (0.76)	23.53 (3.66)	0.76	0.67	0.00^1^	1.00
Median	42.74 (0.92)	22.91 (4.31)	0.72	0.74	0.00^1^	1.00
40 Gy	80.37 (13.53)	0.29 (0.27)	0.54	0.17	0.00^1^	1.00
Left femoral head						
Minimum	25.58 (4.02)	9.30 (2.61)	0.13	0.32	0.00^1^	1.00
Mean	38.48 (3.99)	27.27 (4.06)	0.27	0.27	0.00^1^	0.97
Median	38.55 (6.24)	26.34 (3.82)	0.14	0.68	0.01^1^	0.90
30 Gy	84.13 (14.59)	33.96 (15.61)	0.58	0.24	0.00^1^	1.00
40 Gy	42.86 (18.67)	11.85 (9.02)	0.63	0.56	0.01^1^	0.83
Right femoral head						
Minimum	26.56 (4.06)	9.66 (2.17)	0.79	0.71	0.00^1^	1.00
Mean	39.40 (4.49)	27.46 (2.39)	0.35	0.99	0.00^1^	1.00
Median	38.66 (4.82)	26.78 (2.53)	0.25	0.85	0.00^1^	0.99
20 Gy	100.00^2^ (–)	76.17 (9.51)	–	0.70	0.00^1^	1.00
30 Gy	85.89 (15.91)	38.72 (7.70)	0.23	0.78	0.00^1^	1.00
40 Gy	44.07 (26.77)	10.95 (6.02)	0.88	0.66	0.03^1^	0.66

Variable	Conformal technique	IMRT	Test of normality Shapiro–Wilk		Mann–Whitney test	Observed power *β*
		
	Median (SD)	Median (SD)	Conformal technique	IMRT		
Not normally distributed data						
PTV45						
CN	0.56 (0.12)	0.87 (0.09)	0.06^1^	0.03^1^	0.01^1^	0.95
HTCI	0.56 (0.12)	0.87 (0.10)	0.04^1^	0.03^1^	0.01^1^	0.95
Bladder						
Median	46.85 (0.85)	34.00 (6.87)	0.03^1^	0.21	0.01^1^	0.99
30 Gy	100.00 (1.76)	60.49 (21.37)	0.00^1^	0.33	0.01^1^	0.96
Genitalia						
20 Gy	99.86 (0.40)	68.04 (22.24)	0.01^1^	0.93	0.01^1^	0.88
30 Gy	99.54 (1.87)	12.38 (14.53)	0.00^1^	0.05	0.01^1^	1.00
50 Gy	6.00 (8.79)	0.00 (0.00)	0.03^1^	0.00^1^	0.01^1^	0.54
Left femoral head						
20 Gy	100.00 (0.07)	75.80 (17.60)	0.00^1^	0.56	0.01^1^	0.68

SD, standard deviation; CN, conformation number; HTCI, Healthy Tissue Conformity Index; PTV, planning target volume; Gy, Gray; PTV54, planning target volume receiving 54 Gy; PTV45, planning target volume receiving 45 Gy; IMRT, intensity modulated radiation therapy.

1 Statistically significant (<0.05).

2 All data points equal for this variable.

### Planning target and GTV

The PTV dose–volume comparisons resulted in a significant difference in the PTV54 maximum. The IMRT plan (mean (*M*) = 60.37, standard error (SE) = 0.42) resulted in a significantly higher maximum dose for the PTV54 (*t*-statistic (*t*) (8 (d*f*)) = −2.79, *P* = 0.02) than the conformal technique (*M* = 58.77, SE = 0.40). There was a significant difference between the two planning techniques for the conformity index data. The Healthy Tissue Conformity Index for the PTV45, Mann–Whitney test (U) = 0.00, *z*-score (*z*) = −2.62, *P* = 0.01, effect size (*r*) = −0.83 (conformal, median (Mdn) = 0.56; IMRT, Mdn = 0.87), and PTV54, *t*(8) = −12.12, *P* = 0.00, *r* = 0.97 (conformal, *M* = 0.37, SE = 0.01; IMRT, *M* = 0.76, SE = 0.03) were significantly higher (better) with IMRT compared with the conformal technique. Similarly, for the conformation number, for the PTV45, *U* = 0.00, *z* = −2.62, *P* = 0.01, *r* = −0.83 (conformal, Mdn = 0.56; IMRT, Mdn = 0.87), and PTV54 *t*(8) = −11.15, *P* = 0.00, *r* = 0.97 (conformal, *M* = 0.37, SE = 0.01; IMRT, *M* = 0.75, SE = 0.03) were both significantly higher with the IMRT technique.

### Critical structures

Results for the bladder, external genitalia and femoral heads all indicated that the conformal technique had significantly higher mean or median doses than the IMRT plan (refer to Table 3). There were no statistically significant differences identified between the bladder volume receiving 10 Gy (V10) and maximum data. The largest difference in plans was for the bladder V40 data, *t*(8) = 6.05, *P* = 0.00, *r* = 0.91 (conformal, *M* = 87.18, SE = 4.98; IMRT, *M* = 33.87, SE = 7.27).

For the external genitalia, the largest significant difference in the plans was for the V40 data, *t*(8) = 13.23, *P* = 0.00, *r* = 0.98 (conformal, *M* = 80.37, SE = 6.05; IMRT, *M* = 0.29, SE = 0.12). The largest significant difference for the left and right femoral heads was for the minimum data, *t*(8) = 7.42, *P* = 0.00, *r* = 0.93 (conformal, *M* = 25.58, SE = 1.80; IMRT, *M* = 9.77, SE = 1.14); *t*(8) = 8.58, *P* = 0.00, *r* = 0.95 (conformal, *M* = 26.56, SE = 1.81; IMRT, *M* = 10.31, SE = 0.54).

The small and large bowel data could not be statistically compared due to the limited sample size, and the extreme variation in bowel volume between the cases.

## Discussion

All five patients in this study were female – corresponding with Australian Institute of Health and Welfare (AIHW) data,^21^ where the female to male ratio was 3.7:1 (78.5%:21.5%) diagnosed with anal carcinomas each year (based on data between 1982 and 2008). The patients' age range was identical to Milano et al.^4^; however, the mean and median age were lower in this study. Milano et al.^4^ and Menkarios et al.^11^ both reported patients with T4 staging, yet this study along with Brooks et al.^12^ had no T4 cases. There was a similar distribution of cases throughout the current and previous studies^4^,^11^,^12^ for nodal involvement. There were two research questions stated in the Introduction that were addressed.

### PTV and tumour volume

The Healthy Tissue Conformity Index and the conformation number data revealed IMRT to have superior PTV dosimetry when compared with conformal radiotherapy plans. Brooks et al.^12^ was the only study previously to have used conformity index data when comparing IMRT and conformal treatment for anal carcinomas. Their results concurred that IMRT had better PTV dose coverage compared with conformal.^12^ No study had previously reported conformation number data for IMRT versus conformal dosimetry for anal carcinoma.

The PTV maximum doses were significantly higher for IMRT when compared with conformal radiotherapy; however, this was only a point dose measurement. The maximum dose for conformal radiotherapy was not always located within the PTV (i.e., in some circumstances the maximum dose point was located in the arch of the horseshoe-shaped PTV, therefore, being outside the PTV). This was a drawback of conformal radiotherapy compared with IMRT, where the maximum point dose for the IMRT technique, as well as the biologically significant maximum, was all located within the PTV. It was not possible to compare the biologically significant maximum dose for the two planning techniques as these data were not collected.

### Critical structures

The IMRT plans resulted in significant decreases in the dose received by the surrounding critical structures (bladder, external genitalia and femoral heads). This concurred with previous literature that has shown IMRT to decrease the dose to critical structures.^4^,^11^,^12^,^22^–^24^ The dosimetric findings of this study were similar to those of Brooks et al.^12^ in relation to the critical structures and tumour coverage; the major difference was that this study did not collect data on the healthy tissue volume.

In this study there were no statistically significant differences between the conformal and IMRT plans for the dose received by the small and large bowel; however, Brooks et al.^12^ reported a significant reduction in dose to the bowel for the mean and at V30. Statistical analysis of bowel data in this study was challenging due to the large variation in bowel volume between cases. On a case-by-case basis, the DVH data demonstrated large reductions in bowel dose for the IMRT plans in comparison with the conformal technique; however, this difference was not statistically significant.

General observations suggested that patients who received IMRT appeared to tolerate the treatment better. Toxicity reporting data at the Andrew Love Cancer Centre for this cohort of anal carcinoma cases were incomplete; however, all skin toxicity data were collected where patients with conformal treatment all experienced Grade 3 skin toxicity, whereas those treated with IMRT only experienced Grade 2 skin toxicity.^13^ Of the five cases, no IMRT case required a treatment break; however, two of the three conformal cases required a week break from treatment. It must be noted that the small cohort in this comparison limits its use for making generalizations to the wider treatment population or even comparing with other studies. However, IMRT may improve acute skin toxicity and reduce the need for treatment breaks, but further investigation is required to confirm this.

There were a number of limitations in this study and these are listed below:

1. There were incomplete acute toxicity data available for this cohort of patients and there were no available late toxicity data. The recording of acute and late toxicity data has now been addressed at the Andrew Love Cancer Centre, where all toxicities are reported and these will be used in further investigations in this area to validate the findings of this research.
2. The study consisted of a small sample of patients, limiting the power of statistical analysis. However, this does not detract that there were some statistically significant findings regardless of the small patient cohort. Further research with a larger sample of patients will be required to validate the findings of this study and provide evidence to underpin statistical comparisons where the power was not sufficient in the current study.

For anal canal cases IMRT will be the technique of choice at the Andrew Love Cancer Centre, based on the improved dose conformity, healthy tissue sparing, and increased control over the size and location of hot spots. Although the resources used, including the time taken to plan and treatment anal canal cases with IMRT, were considered at the Andrew Love Cancer Centre, the dosimetric improvements with the potential for decreased toxicities were the focus of this study.

## Conclusion

This study investigated the difference between the Andrew Love Cancer Centre conformal and IMRT plans for anal carcinoma. The results indicated that there was improved dose conformity with IMRT plans and a reduction in dose to the surrounding critical structures (bladder, external genitalia and femoral heads). In addition, observed evidence suggested that patients at the Andrew Love Cancer Centre tolerated IMRT better than the conformal radiotherapy; however, further investigation is required to quantify this. In the future, research in this area will include continuing to collect dosimetric, along with acute and late toxicity data on anal canal cases to validate the findings of this study.
